# A waterborne gastroenteritis outbreak caused by norovirus GII.9[P7] in Guangdong, China

**DOI:** 10.1186/s12879-025-12323-1

**Published:** 2025-12-19

**Authors:** Caixia Li, Yingtao Zhang, Biao Zeng, Qiong Huang, Bixia Ke, Wei Zhang, Hanri Zeng, Jing Lu, Baisheng Li

**Affiliations:** 1 Guangdong Provincial Center for Disease Control and Prevention, Guangdong Workstation for Emerging Infectious Disease Controland Prevention, Chinese Academy of Medical Sciences, 160 Qunxian Road, Guangzhou, China; 2 Guangdong Provincial Institution of Public Health, Guangdong Provincial Center for Disease Control and Prevention, 160 Qunxian Road, Guangzhou, China

**Keywords:** Gastroenteritis, GII.9[P7], Norovirus, Outbreak, Waterborne

## Abstract

**Background:**

Noroviruses are a predominant cause of acute gastroenteritis (AGE) outbreaks globally, many outbreaks are associated with waterborne transmission. However, waterborne AGE outbreaks caused by the GII.9[P7] strain are relatively rare.

**Methods:**

In April 2024, an AGE outbreak occurred among high school students on an educational excursion in Guangdong, China. Feces or anal swabs from clinical cases and asymptomatic canteen staffs, water samples from septic tank and tap water, along with food samples were collected for pathogen detection by real-time RT-PCR, and positive samples were subsequently characterized through gene sequencing analysis.

**Results:**

From 12 April to 14 April 2024, a total of 84 individuals met the case definitions. The cases occurred continuously throughout the excursion without a distinct epidemic peak and the number of cases decreased significantly after the students left on April 13. Norovirus GII was detected in 12 symptomatic cases (12/24) and an asymptomatic food handler (co-infected with rotavirus A,1/7) and all water samples (7/7). The norovirus strain was identified as GII.9[P7] based on phylogenetic analysis, with 100% nucleotide sequence identity among the clinical cases and water samples, implying that the causative agent of the outbreak originates from contaminated drinking water.

**Conclusions:**

This study identified GII.9[P7] norovirus as the causative agent of this outbreak. This was the first reported waterborne outbreak of GII.9[P7] norovirus in China. Our study highlights the necessity of an integrated environmental and clinical case surveillance system for prevention and control of norovirus-associated gastroenteritis outbreak.

## Introduction

Norovirus is a highly contagious pathogen and the primary cause of human non-bacterial acute gastroenteritis (AGE) outbreaks [1]. Owing to its low infectious dose, prolonged viral shedding, and resilience to environmental conditions, norovirus is particularly prone to causing outbreaks in semi-closed settings such as schools, kindergartens, and healthcare facilities [2, 3]. Humans lack long-term immunity and cross-protective immune mechanisms against noroviruses. To date, there are no effective vaccines or specific antiviral therapies available for the prevention or treatment of norovirus-associated diarrhea, which has led to a significant disease burden globally [4].

Norovirus has a positive-sense, single-stranded RNA genome with approximately 7.5kb to 7.7kb in length, it encompasses three open reading frames (ORFs), namely ORF1, ORF2, and ORF3. ORF1 mainly encodes six non-structural proteins, including RNA-dependent RNA polymerase (RdRp), while ORF2 encodes the major structural protein (VP1), and ORF3 encodes the minor structural protein (VP2). VP1 is classified into 10 genogroups (GI-GX), among which GI and GII are the primary genogroups infecting humans. ORF3 encodes the minor structural protein VP2, which plays an important role in the assembly and stability of the viral particles [5, 6]. Norovirus mutates rapidly and undergoes frequent recombination, therefore, molecular monitoring of norovirus is indispensable for epidemiological investigations [7].

Approximately 90% of all global epidemic nonbacterial gastroenteritis outbreaks are estimated to be caused by norovirus [4]. Norovirus spreads commonly via the fecal-oral route, direct person-to-person contact, and indirect pathways including contaminated food, water, or surfaces [8, 9]. Symptomatic patients and asymptomatic carriers serve as the main transmission sources of infection. Norovirus is continuously shed into the environment via the fecal-oral route for several days, leading to community transmission chains. Notably, norovirus exhibits remarkable environmental persistence, it remains infectious in groundwater for at least 61 days and can still be detected after more than 3 years [10]. Despite coordinated endeavors to improve food safety, waterborne outbreaks persist.

Norovirus GII.9[P7] strain has been documented in sporadic gastroenteritis cases [11–13], however, it rarely causes outbreaks. In April 2024, an AGE outbreak occurred among high school students on an educational excursion in Guangdong. Many students exhibited gastrointestinal symptoms such as nausea, vomiting, diarrhea, and abdominal pain. Epidemiological investigations, laboratory pathogen detection, and molecular epidemiological analysis indicated that the incident was caused by a rare norovirus strain, GII.9[P7]. We further explored the genetic differences between current and previously reported GII.9[P7] strains aiming to provide new insights into the genetic diversity and evolution of norovirus, as well as the development of prevention and treatment strategies.

## Methods

### Case definition and specimen collection

According to the National Viral Diarrhea Surveillance Protocol (2021 version), an outbreak was defined as the occurrence of ≥ 5 epidemiologically-linked AGE cases within three days or ≥ 20 epidemiologically-linked AGE cases within a week with at least 2 laboratory-confirmed cases. Epidemiology data was collected from the AGE cases. A cohort of 511 students participated in an educational excursion to a scenic area from April 11 to 13, 2024. An outbreak occurred on the second day (April 12). Clinical specimens, including anal swabs or stool samples, were obtained from both symptomatic cases and asymptomatic kitchen staff members. Food samples were collected on the same day for investigation. The scenic area’s drinking water was supplied by a groundwater well. Notably, no relevant water quality test reports or cleaning and disinfection records were available for review. Furthermore, environmental samples of 5 L each were collected as follows: 1 sample from the groundwater well and 5 from tap water outlets between April 13 and 14, followed by a sample of septic tank effluent on April 15.

### Pretreatment of samples and RNA extraction

After anal swabs or fecal suspension were centrifuged at 8,000 g for 5 min, the supernatant was collected for analysis. For virus detection in water samples, 5 L aliquots were processed by concentration via negatively charged membrane filtration and ultrasonication [14]. This procedure involved an initial 30-minute centrifugation at 4 °C to collect supernatant, followed by the addition of 0.05 M MgCL₂ and HCl to adjust the pH to 3.5-4.0. The acidified solution was then slowly passed through a negatively charged membrane filter (Advantec, Japan). Then the membrane was cut into pieces and sonicated for 2 min in 3% beef extract solution (pH 9.6). The eluate was collected by subsequent centrifugation at 3,000 × g for 30 min. The detection of Salmonella and Shigella from clinical case samples was performed using both bacterial culture and Real-time RT-PCR analysis, as described previously [15]. Retained food samples from April 11th and 5 L water samples were processed for the detection of Norovirus following Microbiology of the food chain Horizontal method for determination of hepatitis A virus and norovirus using real-time RT-PCR (ISO/TS 15216:2017). Total nucleic acid was extracted from 200 µL of the prepared supernatants (from anal swabs or water concentrates) using a commercial RNA extraction kit (Xi’an Tianlong Science & Technology, China), following the manufacturer’s protocol. 100 ml water samples were analyzed for the fecal indicator bacteria, total coliforms and Escherichia coli (E. coli), by Standard Examination Methods for Drinking Water (GB/T 5750 − 2023).

### Real-time RT-PCR detection

All samples were tested for rotavirus A and norovirus with TaqMan realtime RT-PCR using commercially available rotavirus/norovirus (GI and GII) nucleic acid testing kit (Bojie, China) according to the manufacturer’s instructions with Applied Biosystems 7500 Real Time PCR System (Applied Biosystems, United States).

### Genotyping

All positive samples for norovirus were further analyzed by conventional RT-PCR using the SuperScript IV One-Step RT-PCR Kit (Invitrogen, USA). The primers Mon 431/G2SKR were used to amplify the junction region of the partial RdRp and capsid genes [16], generating a 557 base pairs (bp) amplicon spanning nucleotides 4782–5339. Amplification conditions were as follows:10 min of reverse transcription at 50 °C, and then 2 min of initial denaturation at 98 °C. PCR amplification was performed for 40 cycles (98 °C for 10 s, 60 °C for 10 s, and 72 °C for 30 s), and a final elongation at 72 °C for 5 min. Sanger dideoxy sequencing of the PCR products was carried out following 1% agarose gel. The obtained assembly sequences were genotyped via BLAST (http://blast.ncbi.nlm.nih.gov/).

One GII.9[P7]-positive stool sample (Ct < 26) was selected for next-generation sequencing (NGS) sequencing using Illumina Miseq sequencing platform as previous report [17]. The full norovirus genome was amplified by RT-PCR using a Multiple PCR Enrichment Kit for norovirus (Shanghai Biogerm, China), the products were purified using AMPure XP beads (Beckman Coulter, USA) and quantified via a Qubit assay (Invitrogen, USA). Norovirus whole-genome libraries were subsequently prepared using a commercial kit (Shanghai Biogerm, China) according to the manufacturer’s instructions. The sequencing data were assembled via SPAdes-3.15.5 software.

### Sequence analyses

Phylogenetic analysis was performed using MEGA11 software (http://www.megasoftware.net/) employing the neighbor-joining algorithm with 1,000 bootstrap replicates to assess nodal support. Reference sequences of complete or partial GII.9 and GII.P7 genomes were downloaded from NCBI. Multiple sequence alignment was conducted using ClustalW, and BioEdit software was used for amino acid sequence analysis and identification of histo-blood group antigen (HBGA) binding sites [18].

## Results

### Epidemiological investigation

An AGE outbreak occurred in a commercial scenic area during an educational excursion organized for senior high school students. According to the epidemiological investigation data, none of the students or the teachers participating in the educational excursion exhibited symptoms of gastroenteritis at the beginning of the trip. Furthermore, the investigation revealed that they had no contact with individuals experiencing diarrhea and/or vomiting in the days prior to the trip. Following their arrival on April 11, 2024, the onset of symptoms in the first case occurred on April 12, subsequent cases emerged continuously throughout the excursion without a distinct epidemic peak. The number of cases decreased significantly after the students’ departure on April 13, and no new cases were reported after April 15.

Among the 531 participants, 84 cases (15.8%) met the AGE case definition, including 81 students and 3 instructors, with ages ranging from 15 to 28 years and a male-to-female ratio of 0.53:1. The most prevalent clinical symptom was diarrhoea (81/84, 96.4%), followed by vomiting (77/84, 91.7%), abdominal pain (56/84, 66.7%), and fever (51/84, 60.7%). All the cases were recovered within 48–72 h, with no severe complications or deaths reported.

### Etiological investigation

To identify the causative pathogens in this outbreak, a total of 45 samples were collected respectively, including 3 stool samples and 21 rectal swab samples from infection cases, 7 rectal swab samples from asymptomatic canteen staff members, 7 food samples and 7 water samples (1 from septic tank effluent, 1 from groundwater well and 5 from tap water). Pathogenic bacteria Salmonella and Shigella were not identified in any of the human case specimens or samples from canteen staff. Norovirus GII was identified in 2 stool specimens and 10 rectal swabs from the cases. Additionally, 1 asymptomatic canteen staff member was found to be co-infected with Norovirus GII and group A rotavirus (RVA), while all other asymptomatic canteen staff tested negative. No norovirus GII, or RVA were detected in the food samples. Notably, high levels of coliforms and E. coli were detected in water samples from both the well and tap water, suggesting possible faecal contamination. Additionally, all 7 water samples (from the well, septic tank effluent, and tap water) tested positive for NoV GII, GI, and RVA.

**Fig. 1:**
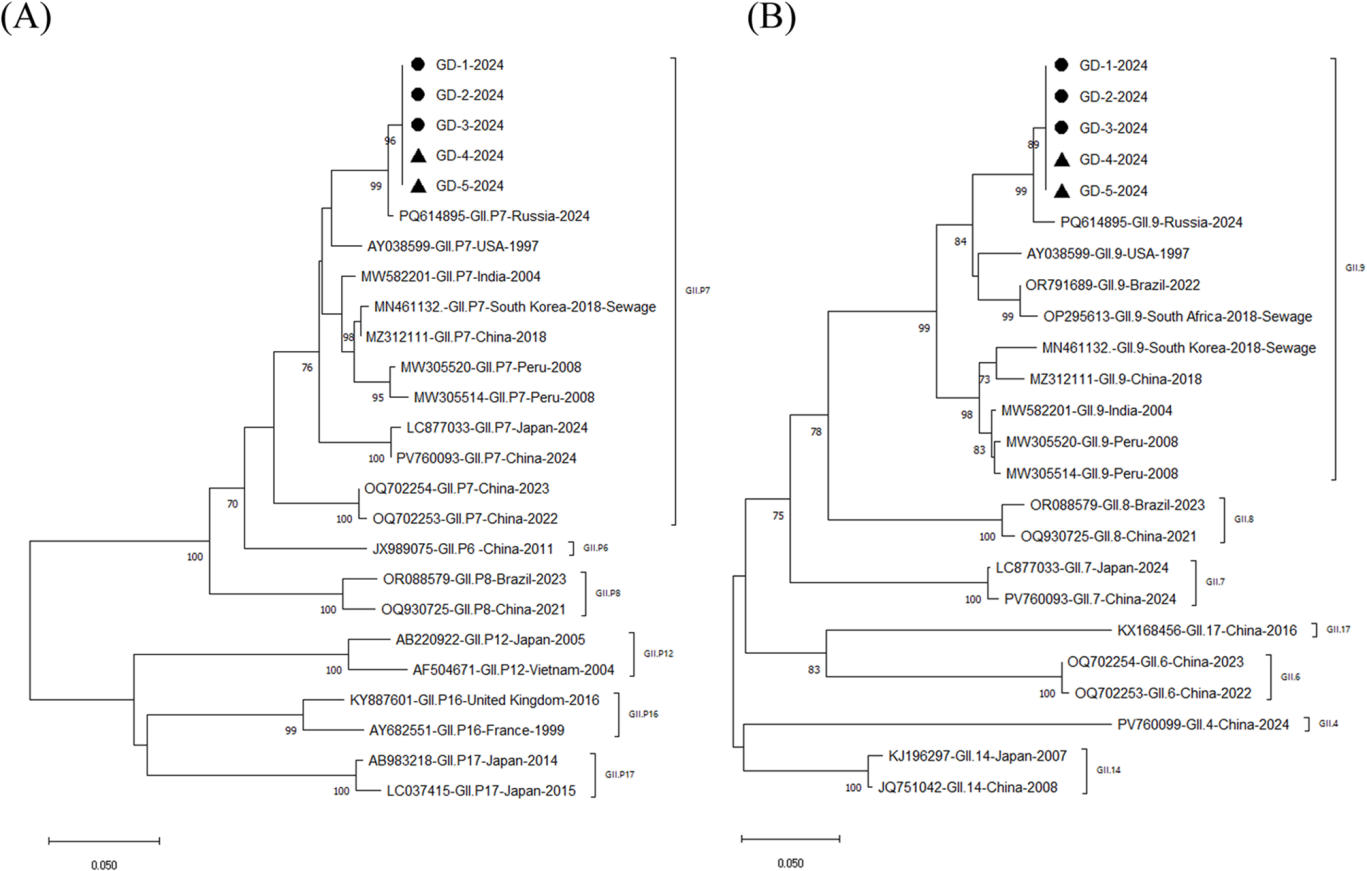
Phylogenetic analysis based on partial nucleic acid sequences of the norovirus GII.9[P7] ORF1 and ORF2 genes. (**A**) Phylogenetic tree constructed using partial ORF1 sequences. (**B**) Phylogenetic tree constructed using partial ORF2 sequences. Both trees were generated by the neighbor-joining method with 1000 bootstrap replicates using MEGA version 11. Bootstrap values > 70% are shown. Reference strains of norovirus genotypes are labeled with their corresponding GenBank accession numbers. The scale bar at the bottom represents genetic distance. Specimens from symptomatic students are marked with black circles, and those from the septic tank and tap water are marked with black triangles

Genotyping was successful for only 5 (21%) of the detected norovirus cases, and the asymptomatic case was not genotyped. Most genotyping failures occurred in samples with Ct values greater than 32. Consequently, we obtained 5 nucleotide sequences, encompassing partial ORF1 and ORF2 regions, from 3 human case samples and 2 environmental samples (from a sewage septic tank and tap water) in this outbreak. The nucleotide sequences isolated from the water samples and the clinical infection cases shared 100% sequence identity with each other. All sequenced strains were genotyped as norovirus GII.9[P7] using the norovirus genotyping tool (https://www.rivm.nl/mpf/typingtool/norovirus/), which was further verified by the result of the phylogenetic analysis. For the partial ORF2 genes, the norovirus strains identified in Guangdong in 2024 clustered together with GII.9 strains isolated from sporadic gastroenteritis cases in Russia (PQ614895), as well as from sewage samples in South Africa (Fig. 1). Consistent with the close cluster of the ORF2 sequences, the ORF1 gene sequences from the Guangdong outbreak were highly associated with GII.P7 strains reported in Russia (PQ614895) and the USA (AY038599). These nucleotide sequences were submitted to GenBank with the accession numbers PX362805-PX362809.

### Analysis of a nearly complete genome sequence

**Fig. 2:**
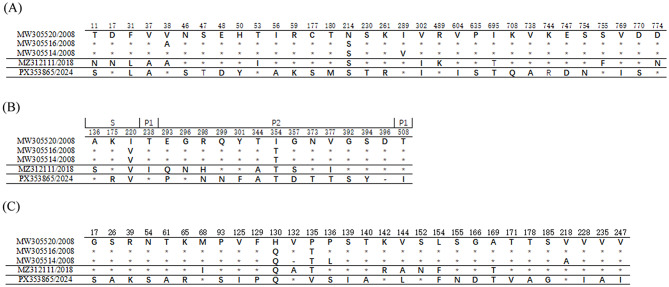
Amino acid variations within the ORF1, ORF2 and ORF3 protein of the GII.9[P7] norovirus strains. (**A**) Mutations in the ORF1 proteins; (**B**) Mutations in the ORF2 proteins; (**C**) Mutations in the ORF3 proteins. conserved variants are shown by *; − is the deletions in the alignment. To date, only four full-length genomic sequences of norovirus GII.9[P7] are available in the NCBI database. The sequence alignment included all five available GII.9[P7] sequences, comprising the four previously reported in NCBI (2008–2018) and the novel strain (PX353865) identified in this study (2024)

One GII.9[P7] nearly complete genomic sequence (accession number: PX353865) was obtained from the stool sample with the maximum norovirus RNA load in this study. There are only a very small number of full-length genomic sequences of norovirus GII.9 in NCBI database, indicating either limited circulation or insufficient surveillance of this genotype. MW305520 strain (collected in Peru, 2008) showed the highest nucleotide sequence identity (88.8%) to the sequence obtained in this study. Guangdong strain and other GII.9[P7] variants exhibited amino acid sequence identities of 97.3–97.5% in ORF1 and 97.2–97.5% in ORF2. In the ORF2 region, several conserved positions differed between the Guangdong outbreak strain and the other four sequences. These differences included amino acid substitutions at the following sites: K175R, Q299N, Y301F, N373T, G392S, S394Y, T508I, along with a single deletion at position 396 (Fig. 2), with the highest variability localized within the P2 subdomain-a region essential for histo-blood group antigen (HBGA) binding interactions. Notably, none of the residues within the known HBGA binding sites (T345, R346, D374, Y389, S439, G440 and H441) [18] exhibited mutations. In contrast, the Guangdong GII.9[P7] strain appeared to diverge most from other GII.9[P7] strains within the minor structural protein ORF3, multiple sequence alignment revealed 25 variable amino acid sites in this region, with pairwise amino acid sequence identities were 89.5%-90.3% to other GII.9[P7] variants, indicating notably higher genetic variation compared to ORF1 and ORF2.

## Discussion

Many studies have reported waterborne norovirus outbreaks caused by contaminated drinking water [19, 20], similar incidents have also been recorded in China [21]. The outbreak occurred among high school students on an educational excursion in a scenic area. As food samples were negative for norovirus, a foodborne outbreak was excluded. The detection of GII norovirus in the patient samples, together with the laboratory findings on the water samples, strongly implies that the causative agent of the outbreak originated from contaminated drinking water. Genotyping analysis further confirmed that the outbreak originated from contaminated drinking water. Group activities during the study tour, combined with reported cases of public vomiting, indicate that limited contact transmission may also have contributed to the spread of the outbreak. Waterborne outbreaks typically cause high attack rates, however, the infection rate here was relatively low, potentially because the prevalent use of bottled water among students, which reduced exposure to the contaminated water, and an effective containment strategy involving the immediate treatment and isolation of cases and the swift departure of all students from the scenic area, thereby eliminating the infection source.

The successful identification in this case may be attributed to the outbreak’s confined setting within a small scenic community. The analysis of water samples collected immediately after the outbreak revealed the presence of fecal indicator bacteria, indicating possible fecal contamination of the groundwater source in the scenic area. Poor sewage management was probably the cause of this waterborne norovirus outbreak. Unfortunately, we did not have a concurrent epidemiological and environmental hygiene investigation for this norovirus outbreak, therefore, we could not delineate the precise pathway of faecal contamination from sewage to the groundwater well. This study enriches our knowledge of norovirus hydro-transmission dynamics in China. In future, we will further strengthen the monitoring of drinking water in scenic areas.

The GII.9[P7] norovirus genotype was first identified during an AGE outbreak in USA in 1997 [12]. A 3290-bp genomic segment of this strain, covering the complete ORF2 coding region, was subsequently deposited in GenBank (AY038599, 2001). Studies have demonstrated that GII.9[P7] can bind to Lewis glycans, and structural characterization indicates it harbors a receptor-binding site analogous to that of the pandemic GII.4 genotype, with fucose playing a key role in mediating interactions [22]. Unlike more prevalent genotypes, GII.9[P7] strains have been rarely reported in surveillance studies, and mainly identified in sporadic infections [23, 24]. An investigation of genotype distribution was conducted among 1085 AGE outpatients from 2019 to 2022 in Brazil, which identified only 1 strain were GII.9[P7] [24]. Zhang ZL et al. first reported the detection of a complete genome sequence of GII.9[P7] norovirus in China from a passenger traveling from India on March 19, 2018 [13]. GII.9 strains have also been detected in wastewater samples from South Africa [25], as well as in oyster samples from Japan [26]. The phylogenetic tree that we constructed from the partial nucleotide sequences of the RdRp and VP1 genes indicated that the 5 strains from this outbreak were all GII.9[P7], implying that GII.9[P7] was the most probable causative agent of this outbreak. Phylogenetic analysis of partial ORF1 and ORF2 segments revealed that the Guangdong strains identified in the study were genetically closer to the Russia 2024 strains (PQ614895) than to the previously identified Chinese strain (MZ312111, 2018). These findings indicate that the Guangdong 2024 strains and Chinese 2018 strains have distinct origins. Due to a lack of timely molecular surveillance data from other regions, the potential origin and spread of this variant in other areas remain largely unknown. Guangdong 2024 strains may originate from neighboring countries or from the local evolution of previously undetected variants circulating in environmental reservoirs.

Notably, waterborne outbreaks associated with GII.9[P7] norovirus remain scarce worldwide. This study reports the first documented waterborne outbreak caused by the norovirus GII.9[P7] strain in China. Although norovirus GII.9 has been detected in wastewater in Shandong [27] and Guangzhou [14], clinical cases remain rare, with limited GenBank sequences and no reported infections in Guangdong to date, possibly due to asymptomatic carriage or mild clinical symptoms that do not require hospitalization, which was difficult to be captured by clinical surveillance systems. While previous research has established the importance of surveillance for norovirus waterborne outbreaks, our study reinforces the importance of continuous surveillance to detect emerging subtypes geographically. It also advocates for the integration of environmental surveillance as a key complementary measure in public health strategies.

Although the divergence between the Guangdong strain and related strains identified in different geographic regions suggests that the virus is undergoing progressive evolution, the HBGA binding sites were conserved in GII.9[P7] strain. No mutations were found in the known HBGA binding site residues. However, other substitutions were identified in the predicted P2 domain [18] (amino acids 279 to 405 of the VP1 protein). Further study should be employed to find whether these mutations in the P2 domain contribute to viral adaptation of the outbreak of GII.9[P7] strains in Guangdong 2024.

Within the ORF3 minor structural protein, 25 variable amino acid residues were identified, and the Guangdong GII.9[P7] strain showed the highest divergence from other GII.9[P7] strains in this region. In mammalian cells, VP2 increased both the expression level and stability of VP1 by protecting it from disassembly and protease degradation [5], whether sequence hypervariability in VP2 exerts analogous effects on norovirus GII.9 strains remains to be clarified. However, given that only a limited number of full-length genomic sequences have been characterized as GII.9 references in GenBank to date, they may not fully represent the diversity of circulating norovirus strains. Continuous genetic and antigenic variation in noroviruses may facilitate their sustained circulation in human populations, with multiple genotypes exhibiting epidemic potential [28]. Although GII.9[P7] strains primarily infect humans only sporadically, continuous monitoring of their sequence variations remains essential.

Our study has several limitations. Firstly, although our laboratory data strongly supports a waterborne origin for the outbreak, we could not definitively confirm the exact contamination point due to the absence of mapping of water and sewage systems and incomplete records of the breakage incident. Secondly, the relatively small number of samples collected may have limited the detection of the full spectrum of causative pathogens. Another significant limitation is the lack of a temporal sequence of epidemiological events, which precludes in-depth analysis of transmission dynamics.

## Conclusions

In this study, we report the first waterborne outbreak of acute gastroenteritis caused by a norovirus GII.9[P7] strain in China. Additionally, we successfully obtained the nearly complete genome sequence of this strain, which has seldom been documented in China. Previous studies have highlighted the high sensitivity of environmental surveillance in tracing the transmission routes of specific viral strains [12, 26]. Our findings underscore the importance of enhancing integrated surveillance strategies that combine environmental water monitoring with clinical case data from sentinel hospitals. Such an approach is critical for offering valuable insights into the emergence of norovirus strains possessing relatively rare genotypes.

## Data Availability

The partial and complete genome sequences of the norovirus strains were deposited in the GenBank database with the accession numbers PX362805- PX362809 and PX353865.

## Abbreviations

AGE: Acute gastroenteritis
ORF: Open reading frames
UTRs: Untranslated regions
RdRp: RNA-dependent RNA polymerase
GI: Genogroup I
GII: Genogroup II
RT-PCR: Reverse transcription polymerase chain reaction

## References

[1] Winder N, Gohar S, Muthana M, Norovirus. An overview of virology and preventative measures. Viruses. 2022;14(12):2811. 36560815 10.3390/v14122811 PMC9781483

[2] White PA. Evolution of norovirus. Clin Microbiol Infect. 2014 Aug;20(8):741–5. 10.1111/1469-0691.12746 24980204

[3] Hall AJ, Wikswo ME, Manikonda K, et al. Acute gastroenteritis surveillance through the National outbreak reporting System, united States. Emerg Infect Dis. 2013;19(8):1305–9. 23876187 10.3201/eid1908.130482 PMC3739540

[4] Ahmed SM, Hall AJ, Robinson AE, et al. Global prevalence of Norovirus in cases of gastroenteritis: a systematic review and meta-analysis. Lancet Infect Dis. 2014;14(8):725–30. 24981041 10.1016/S1473-3099(14)70767-4 PMC8006533

[5] Bertolotti-Ciarlet A, Crawford SE, Hutson AM, et al. The 3’ end of Norwalk virus mRNA contains determinants that regulate the expression and stability of the viral capsid protein VP1: a novel function for the VP2 protein. J Virol. 2003;77(21):11603–15. 14557646 10.1128/JVI.77.21.11603-11615.2003 PMC229252

[6] Vongpunsawad S, Venkataram Prasad BV, Estes MK. Norwalk virus minor cap Sid protein VP2 associates within the VP1 shell domain. J Virol. 2013;87(9):4818–25. 23408637 10.1128/JVI.03508-12 PMC3624303

[7] van Beek J, de Graaf M, Al-Hello H, et al. Molecular surveillance of norovirus, 2005-16: an epidemiological analysis of data collected from the NoroNet network. Lancet Infect Dis. 2018;18(5):545–53. 29396001 10.1016/S1473-3099(18)30059-8

[8] Hassard F, Sharp JH, Taft H, et al. Critical review on the public health impact of Norovirus contamination in shellfish and the environment: A UK perspective. Food Environ Virol. 2017;9(2):123–41. 28176295 10.1007/s12560-017-9279-3 PMC5429388

[9] Miura T, Gima A, Akiba M. Detection of Norovirus and rotavirus present in suspended and dissolved forms in drinking water sources. Food Environ Virol. 2019;11(1):9–19. 30560490 10.1007/s12560-018-9361-5

[10] Seitz SR, Leon JS, Schwab KJ, et al. Norovirus infectivity in humans and persistence in water. Appl Environ Microbiol. 2011;77(19):6884–8. 21856841 10.1128/AEM.05806-11 PMC3187119

[11] Jiang X, Zhong WM, Farkas T, et al. Baculovirus expression and antigenic Charac terization of the capsid proteins of three Norwalk-like viruses. Arch Virol. 2002;147(1):119–30. 11855626 10.1007/s705-002-8306-5

[12] Gelaw A, Pietsch C, Mann P, et al. Molecular detection and characterisation of sapoviruses and Noroviruses in outpatient children with diarrhoea in Northwest Ethiopia. Epidemiol Infect. 2019;147:e218. 31364546 10.1017/S0950268819001031 PMC6625200

[13] Zhang Z, Liu D, Zhang Z, et al. Complete genome sequence of GII.9 Norovirus. Arch Virol. 2022;167(1):249–53. 34718885 10.1007/s00705-021-05257-x PMC8556859

[14] Lu J, Peng J, Fang L, et al. Capturing Noroviruses Circulating in the population: sewage surveillance in Guangdong, China (2013–2018). Water Res. 2021;196:116990. 33725645 10.1016/j.watres.2021.116990

[15] Chaudhary R, Dahal M, Gautam S, et al. Improved diagnosis of Gastrointestinal infections using a semi-automated multiplex real-time PCR for detection of enteropathogens[J]. J Med Microbiol. 2021;70(9):001367. 10.1099/jmm.0.001367 34516365

[16] Kojima S, Kageyama T, Fukushi S, et al. Genogroup-specific PCR primers for de tection of Norwalk-like viruses. J Virol Methods. 2002;100(1–2):107–14. 11742657 10.1016/s0166-0934(01)00404-9

[17] Chen Q, Ma J, Gao L, et al. Determination and analysis of whole genome sequence of Recombinant GII.6[P7] Norovirus in Ningxia, China. Infect Genet Evol. 2023;115:105499. 37734510 10.1016/j.meegid.2023.105499

[18] Tan M, Xia M, Chen Y, et al. Conservation of carbohydrate binding interfaces: evidence of human HBGA selection in Norovirus evolution. PLoS ONE. 2009;4(4):e5058. 19337380 10.1371/journal.pone.0005058 PMC2660415

[19] Bitler EJ, Matthews JE, Dickey BW, et al. Norovirus outbreaks: a systematic review of commonly implicated transmission routes and vehicles. Epidemiol Infect. 2013;141(8):1563–71. 23433247 10.1017/S095026881300006X PMC3742101

[20] Ekundayo TC, Igere BE, Oluwafemi YD, et al. Human Norovirus contamination in water sources: A systematic review and meta-analysis. Environ Pollut. 2021;291:118164. 34534825 10.1016/j.envpol.2021.118164

[21] Yu F, Jiang B, Guo X, et al. Norovirus outbreaks in China, 2000–2018: A systematic review. Rev Med Virol. 2022;32(6):e2382. 35946340 10.1002/rmv.2382

[22] Chen Y, Tan M, Xia M, et al. Crystallography of a Lewis-binding norovirus, eluci Dation of strain-specificity to the polymorphic human histo-blood group antigens. PLoS Pathog. 2011;7(7):e1002152. 21811409 10.1371/journal.ppat.1002152 PMC3141052

[23] Tohma K, Lepore CJ, Martinez M, et al. Genome-wide analyses of human no roviruses provide insights on evolutionary dynamics and evidence of coexisting viral populations evolving under recombination constraints. PLoS Pathog. 2021;17(7):e1009744. 34255807 10.1371/journal.ppat.1009744 PMC8318288

[24] Cantelli CP, Tavares GCL, Sarmento SK, et al. Assessment of gastroenteric viruses in marketed bivalve mollusks in the tourist cities of Rio de Janeiro, Brazil, 2022. Viruses. 2024;16(3):317. 38543684 10.3390/v16030317 PMC10974528

[25] Mabasa VV, van Zyl WB, Ismail A, et al. Multiple novel human Norovirus re combinants identified in wastewater in Pretoria, South Africa by Next-Generation sequencing. Viruses. 2022;14(12):2732. 36560736 10.3390/v14122732 PMC9788511

[26] Imamura S, Kanezashi H, Goshima T, et al. Effect of high pressure processing on a wide variety of human Noroviruses naturally present in Aqua-Cultured Japanese oysters. Foodborne Pathog Dis. 2018;15(10):621–6. 30117743 10.1089/fpd.2018.2444

[27] Wang S, Xu M, Lin X, et al. Detection of human Noroviruses in sewage by next generation sequencing in Shandong Province, 2019–2021. Virol J. 2025;22(1):18. 39871378 10.1186/s12985-025-02638-5 PMC11773704

[28] Donaldson EF, Lindesmith LC, Lobue AD, et al. Norovirus pathogenesis: mecha Nisms of persistence and immune evasion in human populations. Immunol Rev. 2008;225:190–211. 18837783 10.1111/j.1600-065X.2008.00680.x

